# Trends in asthma and pneumonia-related mortality in the United States: a CDC wonder database analysis (1999–2023)

**DOI:** 10.3389/fmed.2026.1736476

**Published:** 2026-01-27

**Authors:** Junsheng Jiang, Lina Qi, Shenggang Ding

**Affiliations:** 1 Department of Pediatrics, The First Affiliated Hospital of Anhui Medical University, Hefei, China; 2 Department of Pediatrics, The First People's Hospital of Linping District, Hangzhou, China; 3 Beijing Children's Hospital, Capital Medical University, China National Clinical Research Center of Respiratory Disease, Beijing, China

**Keywords:** age-adjusted mortality rates, asthma, CDC, pneumonia, the United States

## Abstract

**Background:**

This study seeks to investigate mortality trends associated with the simultaneous occurrence of asthma and pneumonia among U.S over the period from 1999 to 2023.

**Methods:**

CDC WONDER was used to identify asthma and pneumonia related deaths that occurred within the United States from 1999 to 2023. Crude and age-adjusted mortality rates (AAMR) were calculated, as well as annual percent change and weighted average annual percent change with 95% confidence intervals for the AAMRs. The Joinpoint Regression Program was used to determine trends in mortality within the study period. Joinpoint regression analysis was employed to determine annual percentage changes (APCs) and assess statistical significance (*P* < 0.05).

**Results:**

From 1999 to 2023, male patients demonstrated greater mortality rates from pneumonia and asthma compared to females. When stratified by race and ethnicity, Black patients had the highest AAMR over the study period at 29.21 per 100,000 people in 1999, as well as the most significant reduction in AAMR to 13.91 per 100,000 people in 2023. Additionally, AAMRs were consistently higher in rural areas compared to urban locations. By age group, patients aged 85+ had the highest overall crude mortality rate at 747.90 per 100,000 people in 1999, with the lowest rate in ages 5–14 at 0.51 per 100,000 people in 1999.

**Conclusions:**

This study highlight epidemiological differences in asthma- and pneumonia-related death. Significant disparities in mortality rates were noted in older-aged, male, Black, and rural patients.

## 1 Introduction

Respiratory diseases remain a leading cause of morbidity and mortality globally, imposing substantial burdens on healthcare systems and public health resources (1). Among these, asthma and pneumonia stand out as two interconnected yet distinct conditions: asthma is a chronic inflammatory disorder of the airways characterized by recurrent episodes of wheezing, dyspnea, and airflow limitation, while pneumonia is an acute lower respiratory tract infection caused by bacteria, viruses, or fungi (2, 3). This interconnection is clinically evident: for instance, asthmatic patients with chronic airway inflammation and impaired mucus clearance are 2–3 times more likely to develop community-acquired pneumonia than non-asthmatic individuals (4), particularly when asthma is poorly controlled. Conversely, viral pneumonia (e.g., influenza-induced pneumonia) can directly irritate airway epithelial cells, triggering immune responses that exacerbate asthma symptoms—with up to 40% of severe asthma exacerbations in adults linked to preceding respiratory infections (5). These examples highlight their bidirectional influence while preserving their distinct pathological features: asthma is a chronic, non-infectious condition, whereas pneumonia is an acute infectious process. Together, these conditions contribute to a significant share of respiratory-related deaths worldwide, with preventable cases accounting for a notable proportion of this burden (6).

In the United States, asthma and pneumonia continue to pose persistent public health challenges (7). For asthma, despite decades of advances in diagnosis and management, the disease remains highly prevalent: in 2022, approximately 8.2% of the U.S. population reported a lifetime asthma diagnosis, with 42.4% of these individuals experiencing at least one asthma attack in the past year (8). Asthma mortality has shown a downward trend since the late 1990s—declining from 1.7 per 100,000 individuals in 1999 to 1.0 per 100,000 by 2010, and stabilizing through 2022—though disparities persist among older adults (aged ≥65 years), racial/ethnic minorities, and rural residents (8, 9). In 2021 alone, 3,517 Americans died from asthma, translating to a mortality rate of 10.6 per million individuals (8); these deaths are often linked to undertreatment, coexisting chronic conditions, or delayed intervention during acute exacerbations (9).

In contrast, pneumonia remains a leading cause of acute respiratory death in the U.S., with high-risk groups (older adults, young children, individuals with chronic comorbidities, and those with weakened immune systems) bearing the brunt of mortality (10, 11). The COVID-19 pandemic (2020–2022) further amplified pneumonia-related mortality, as severe acute respiratory syndrome coronavirus 2 frequently leads to secondary bacterial or viral pneumonia, and pandemic-related disruptions to healthcare access may have delayed treatment for non-COVID pneumonia cases (12, 13). Even beyond the pandemic, pneumonia mortality trends have been shaped by factors such as vaccine uptake, antimicrobial resistance, and socioeconomic barriers to care—highlighting the need for updated analyses to capture post-pandemic recovery patterns (14).

Notably, the relationship between asthma and pneumonia adds complexity to their mortality burdens. Asthma patients, especially those with poorly controlled disease or frequent exacerbations, have impaired airway clearance and chronic inflammation, increasing their risk of developing pneumonia (15). Conversely, pneumonia can trigger severe asthma exacerbations, leading to acute respiratory failure and death—creating a “bidirectional” risk loop. For example, older adults with uncontrolled asthma are not only more likely to develop pneumonia but also have a 30% higher risk of mortality from pneumonia-related exacerbations compared to their non-asthmatic peers (16); similarly, individuals with low socioeconomic status, who often face barriers to timely care, experience a compounded risk of both conditions progressing to severe outcomes. Despite this interdependence, most prior U.S. studies have analyzed asthma and pneumonia mortality in isolation, focusing on single conditions or limited timeframes (5, 10, 17). This gap limits understanding of how these two conditions interact to drive respiratory mortality trends, particularly in the context of recent public health crises and evolving healthcare practices.

To address these limitations, we conducted a population-based analysis using data from the CDC WONDER database. Our study aims to: (1) describe trends in asthma-related and pneumonia-related mortality in the U.S. from 1999 to 2023, encompassing the pre-pandemic, pandemic, and early post-pandemic periods; (2) examine disparities in mortality rates by demographic factors (age, sex, race) and geographic regions (census regions, urban/rural status). By integrating both asthma and pneumonia, our findings will provide a critical update on respiratory health outcomes, inform targeted public health interventions, and guide strategies to reduce preventable respiratory deaths.

## 2 Methods

### 2.1 Study design and period

A retrospective epidemiological analysis was performed to characterize trends in asthma and pneumonia-related mortality in the United States over a 24-year period (1999–2023). Our study was exempt from institutional review board approval, as we utilized a de-identified government-provided public-use dataset in accordance with Strengthening the Reporting of Observational Studies in Epidemiology (STROBE) guidelines.

### 2.2 Data source and case definition

#### 2.2.1 Database selection

Mortality data were retrieved from the CDC WONDER database. This database aggregates cause-of-death information from death certificates for all 50 U.S. states and the District of Columbia, with established utility in prior analyses of asthma and pneumonia mortality trends due to its long-term coverage, population-level scope, and adherence to standardized data collection protocols (18–21).

#### 2.2.2 Case identification

Asthma and pneumonia-related deaths were defined using the ICD-10 J45, J46 and J12-18. Only deaths where asthma and pneumonia was documented as the underlying cause of death were included.

## 3 Data abstraction

Data were extracted from the CDC WONDER database using predefined criteria to capture key variables associated with asthma and pneumonia -related mortality: year of death, annual population size, state and U.S. Census region of residence, place of death, urban-rural classification, age at death, sex, and race/ethnicity.

Place of death was categorized as medical facilities (including inpatient units, outpatient clinics, emergency rooms, death on arrival, and cases with unknown medical facility status), home, hospice facility, nursing home/long-term care, or other unclassified locations. Age at death was stratified into four groups −1–14, 15–44, 45–64, 65–74, 75–84 and ≥85 years—consistent with age group cutoffs used in prior asthma mortality trend analyses (18). Sex was dichotomized as male or female, while race/ethnicity was classified into four mutually exclusive groups: Non-Hispanic (NH) White, NH Black or African American, NH Asian or Pacific Islander, and Hispanic or Latino.

Urban-rural classification was determined using the 2013 National Center for Health Statistics (NCHS) scheme, which relies on 2010 U.S. Census data to categorize counties into three tiers: urban (large metropolitan areas with population ≥1 million), suburban (medium/small metropolitan areas with population 50,000–999,999), and rural (nonmetropolitan areas with population < 50,000) (22). U.S. Census regions were defined per the U.S. Census Bureau's standard classifications: Northeast, Midwest, South, and West.

## 4 Statistical analysis

To evaluate trends in asthma and pneumonia-related mortality, two core mortality metrics were computed per 100,000 population for the 1999–2020 period, stratified by year, race/ethnicity, sex, age group, U.S. Census region, state, and geographical density (urban/suburban/rural).

Age-adjusted mortality rates (AAMRs) were calculated with 95% confidence intervals (CIs) by standardizing asthma and pneumonia-related death counts to the age distribution of the 2,000 U.S. Standard Population (23)—a critical step to eliminate confounding by age structure differences across subgroups and time points. Crude mortality rates (CMRs) were also computed annually, defined as the total number of asthma and pneumonia-related deaths divided by the corresponding annual U.S. population size for each stratum.

The Joinpoint Regression Program was used to identify trends in mortality by fitting log-linear regression models based on the Poisson distribution assumption—appropriate for count-based mortality data. This approach captures variations in mortality over time by modeling the natural logarithm of death counts as a linear function of time, with the Poisson distribution accounting for the discrete and rare nature of the outcome variable. The Monte Carlo permutation test (1,000 permutations) was applied to establish 95% CIs for the line segments connecting statistically significant trend changes. It is a non-parametric statistical test that assesses the significance of trend changes by randomly permuting the observed mortality data to generate a distribution of expected outcomes. By comparing the observed APCs to this distribution, we can determine whether the detected trend shifts are unlikely to occur by chance. This approach is particularly robust for analyzing time-series mortality data with potential non-linear trends, as it avoids assumptions about the underlying data distribution. This method detected meaningful temporal shifts in AAMRs by fitting log-linear regression models to capture variations in mortality over time. APCs were categorized as increasing or decreasing based on whether the slope of mortality change differed significantly from zero, assessed via two-tailed *t*-tests. Statistical significance was set at a *P*-value < 0.05.

## 5 Result

### 5.1 Overall mortality trends (1999–2023)

**Figure 1:**
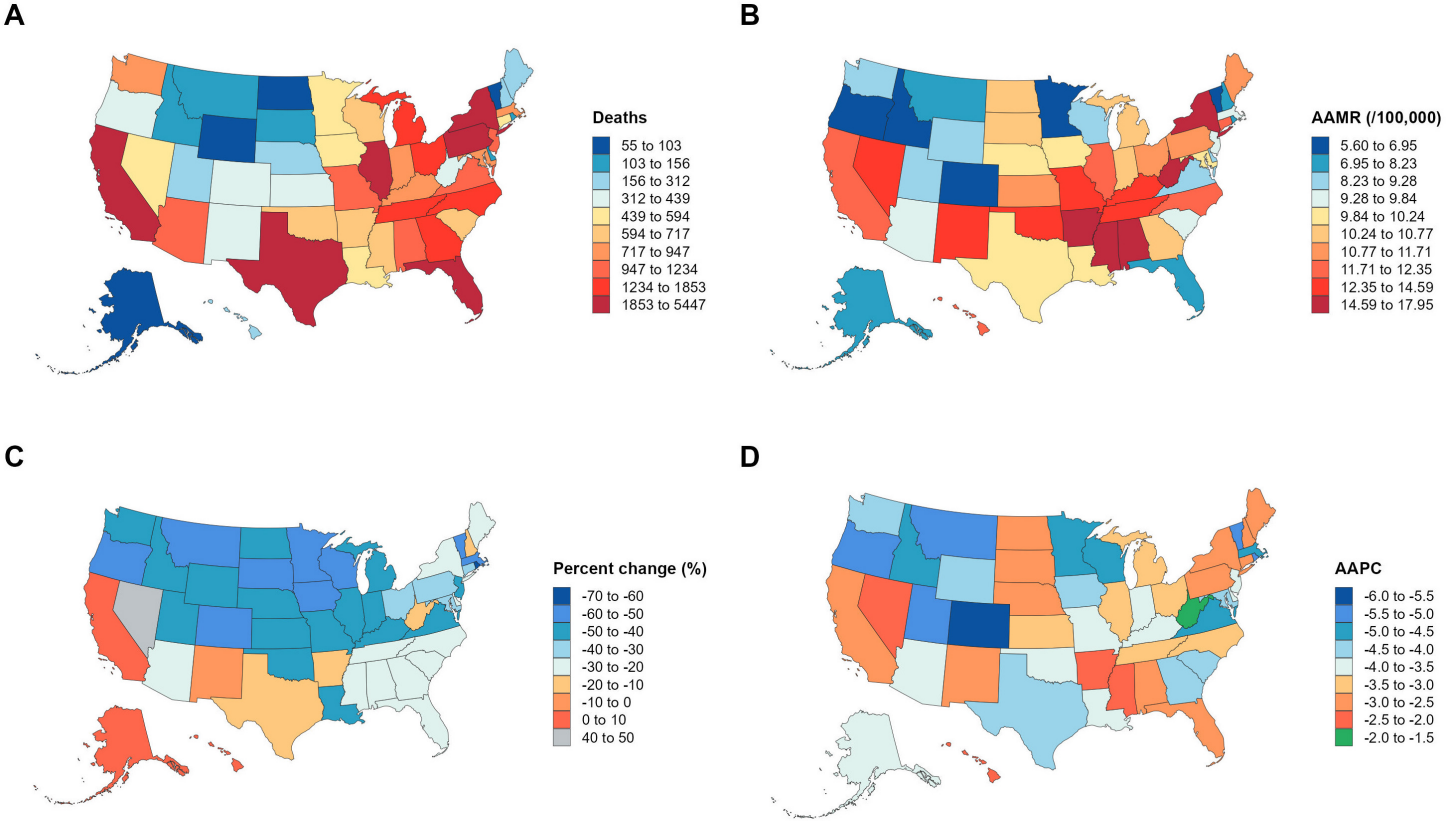
U.S. asthma and pneumonia-related mortality trends (1999–2023): **(A)** Total annual deaths; **(B)** Age-adjusted mortality rate (AAMR) per 100,000 population; **(C)** Percent change in deaths; **(D)** Average annual percent change (AAPC) in AAMR.

From 1999 to 2023, the total number of asthma- and pneumonia-related deaths in the United States decreased significantly, with 66,720 deaths recorded in 1999 and 44,835 deaths in 2023, representing a 32.80% reduction over the 24-year period (Table 1). Consistent with the declining death count, the AAMR also dropped substantially, from 24.58 per 100,000 population (95% CI: 24.40–24.77) in 1999 to 10.82 per 100,000 population (95% CI: 10.72–10.92) in 2023. The average annual percent change (AAPC) in AAMR was −3.57% (95% CI: −3.80 to −3.33, *P* < 0.05), indicating a steady and statistically significant downward trend in overall mortality due to these conditions during the study period (Figure 1).

**Table 1:** Asthma and pneumonia deaths and AAMR in the United States from 1999 to 2023 and their changing trends.

**Characteristic**	**Deaths**			**AAMR**			
	**1999**	**2023**	**Percent change (%)**	**1999 (95% CI)**	**2023 (95% CI)**	**AAPC (95% CI)**	***P***
Total	66,720	44,835	−32.80	24.58 (24.40–24.77)	10.82 (10.72–10.92)	−3.57 (−3.80 to −3.33)	0.001
**Sex**							
Female	37,985	21,954	−42.20	21.93 (21.71–22.15)	9.38 (9.26–9.51)	−3.67 (−3.92 to −3.42)	0.001
Male	28,735	22,880	−20.38	29.24 (28.89–29.59)	12.68 (12.51–12.85)	−3.41 (−4.28 to −2.52)	0.001
**Census region**							
Northeast	14,985	9,216	−38.50	25.78 (25.36–26.19)	11.95 (11.70–12.20)	−3.21 (−3.51 to −2.92)	0.001
Midwest	16,791	9,150	−45.51	25.41 (25.03–25.79)	10.54 (10.32–10.76)	−3.38 (−3.63 to −3.14)	0.012
South	23,560	16,863	−28.43	25.05 (24.73–25.37)	10.71 (10.54–10.87)	−3.53 (−3.75 to −3.31)	0.008
West	11,384	9,605	−15.63	21.43 (21.03–21.82)	10.24 (10.04–10.45)	−3.33 (−4.32 to −2.33)	0.001
**Race**							
Hispanic	2,521	4,045	60.45	20.28 (19.43–21.12)	9.35 (9.06–9.65)	−3.84 (−4.19 to −3.48)	0.001
NH Black	6,863	5,783	−15.74	29.21 (28.50–29.92)	13.91 (13.55–14.28)	−3.10 (−3.42 to −2.78)	0.001
NH White	55,768	32,248	−42.17	24.13 (23.93–24.33)	10.67 (10.55–10.79)	−3.33 (−4.24 to −2.40)	0.001
NH Other	1,298	2,586	99.23	20.34 (19.18–21.51)	8.87 (8.52–9.21)	−4.06 (−4.72 to −3.39)	0.001
**Urbanization** ^a^							
Metropolitan	52,873	35,215	−33.40	24.11 (23.90–24.32)	12.30 (12.18–12.42)	−3.65 (−3.96 to −3.35)	0.018
Nonmetropolitan	13,847	9,620	−30.53	26.48 (26.04–26.93)	14.75 (14.45–15.06)	−2.96 (−3.24 to −2.67)	0.026
**Age groups** ^b^							
< 1 year	312	132	−57.69	8.22 (7.31–9.13)	3.62 (3.00–4.23)	−3.56 (−4.40 to −2.71)	0.001
1–4 years	145	127	−12.41	0.95 (0.79–1.10)	0.85 (0.71–1.00)	−0.60 (−3.61 to 2.49)	0.654
5–14 years	208	156	−25.00	0.51 (0.44–0.58)	0.38 (0.32–0.44)	−0.65 (−1.29 to −0.01)	0.001
15–24 years	350	322	−8.00	0.90 (0.81–1.00)	0.73 (0.65–0.81)	−0.93 (−1.49 to −0.36)	0.001
25–34 years	592	649	9.63	1.47 (1.35–1.59)	1.43 (1.32–1.53)	0.11 (−0.33 to 0.56)	0.563
35–44 years	1,494	1,150	−23.03	3.31 (3.15–3.48)	2.59 (2.44–2.74)	−0.86 (−1.59 to −0.13)	0.001
45–54 years	2,278	1,947	−14.53	6.23 (5.97–6.48)	4.81 (4.59–5.02)	−0.80 (−1.18 to −0.42)	0.001
55–64 years	3,137	4,647	48.14	13.19 (12.73–13.65)	11.10 (10.78–11.42)	−0.40 (−1.17 to 0.37)	0.754
65–74 years	7,470	8,786	17.62	40.56 (39.64–41.48)	25.33 (24.80–25.86)	−2.02 (−2.57 to −1.47)	0.001
75–84 years	19,666	12,206	−37.93	160.87 (158.62–163.12)	66.45 (65.27–67.63)	−3.84 (−4.07 to −3.62)	0.001
85+ years	31,068	14,712	−52.65	747.90 (739.59–756.22)	237.48 (233.65–241.32)	−4.95 (−6.20 to −3.69)	0.001

^a^In the context of urbanization, the 2023 AAMR data was substituted with that from 2020, and the AAPC was calculated based on the period from 1999 to 2020.

^b^For the age groups, the crude mortality rate was used as a substitute for AAMR, and the AAPC was computed based on the crude mortality rate.

AAMR, age-adjusted mortality rate; CI, confidence interval; AAPC, average annual percent change; NH, non-Hispanic.

### 5.2 Mortality trends by sex

Marked differences in mortality trends were observed between males and females. In 1999, males had a higher AAMR (29.24 per 100,000; 95% CI: 28.89–29.59) than females (21.93 per 100,000; 95% CI: 21.71–22.15), and this disparity persisted in 2023, with males still exhibiting a higher AAMR (12.68 per 100,000; 95% CI: 12.51–12.85) compared to females (9.38 per 100,000; 95% CI: 9.26–9.51; Table 1).

**Figure 2:**
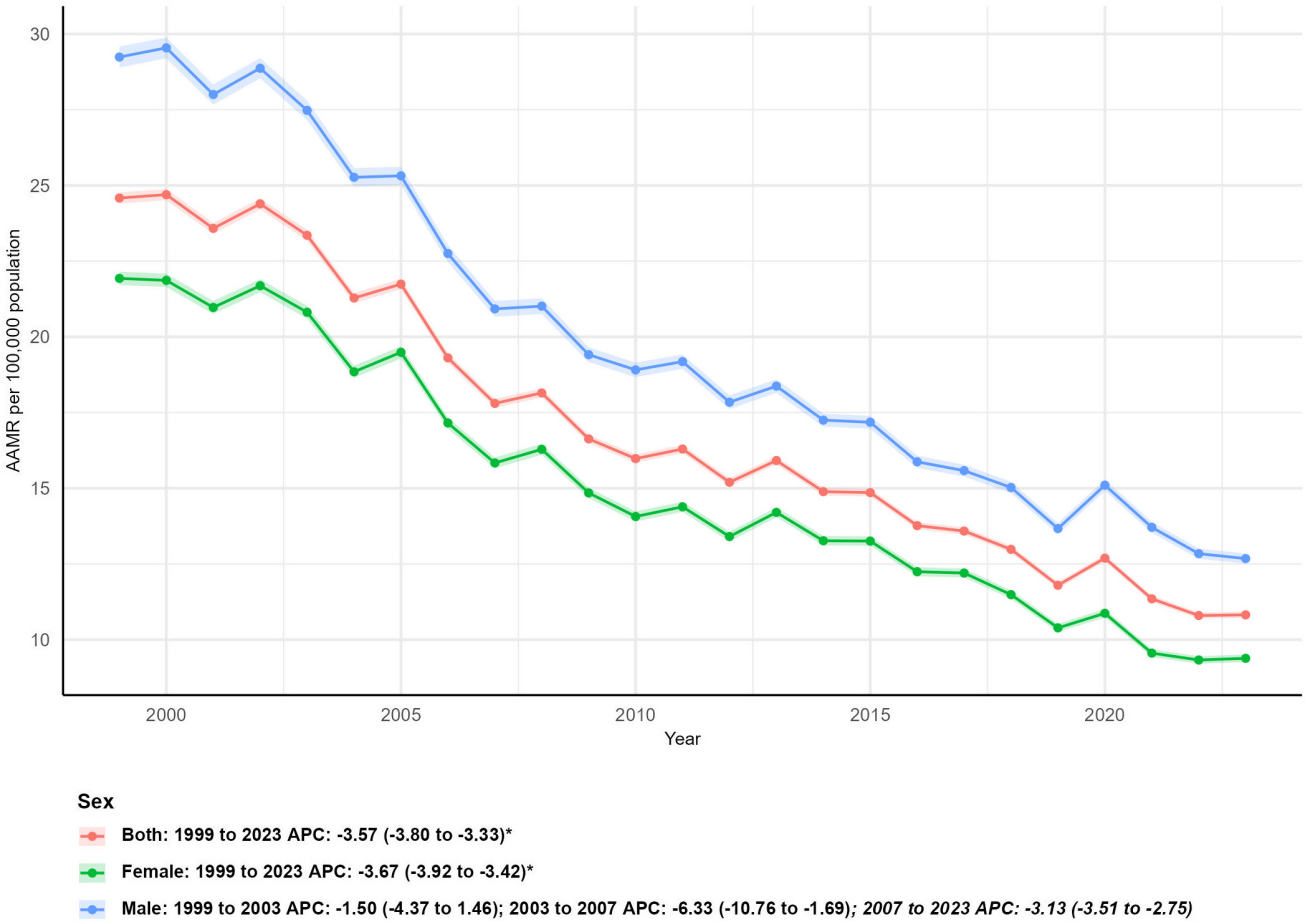
Sex-specific age-adjusted mortality rates (AAMR) for asthma and pneumonia in the U.S. (1999–2023), with annual percent changes (APCs) for females, males, and the overall population.

Over the study period, females experienced a more substantial reduction in both death counts and AAMR: female deaths decreased by 42.20% (from 37,985 in 1999 to 21,954 in 2023), with an AAPC of −3.67% (95% CI: −3.92 to −3.42, *P* < 0.05). In contrast, males had a smaller 20.38% reduction in deaths (from 28,735 in 1999 to 22,880 in 2023) and a slightly lower AAPC of −3.41% (95% CI: −4.28 to −2.52, ^*^P^*^ < 0.05; Figure 2).

### 5.3 Mortality trends by census region

**Figure 3:**
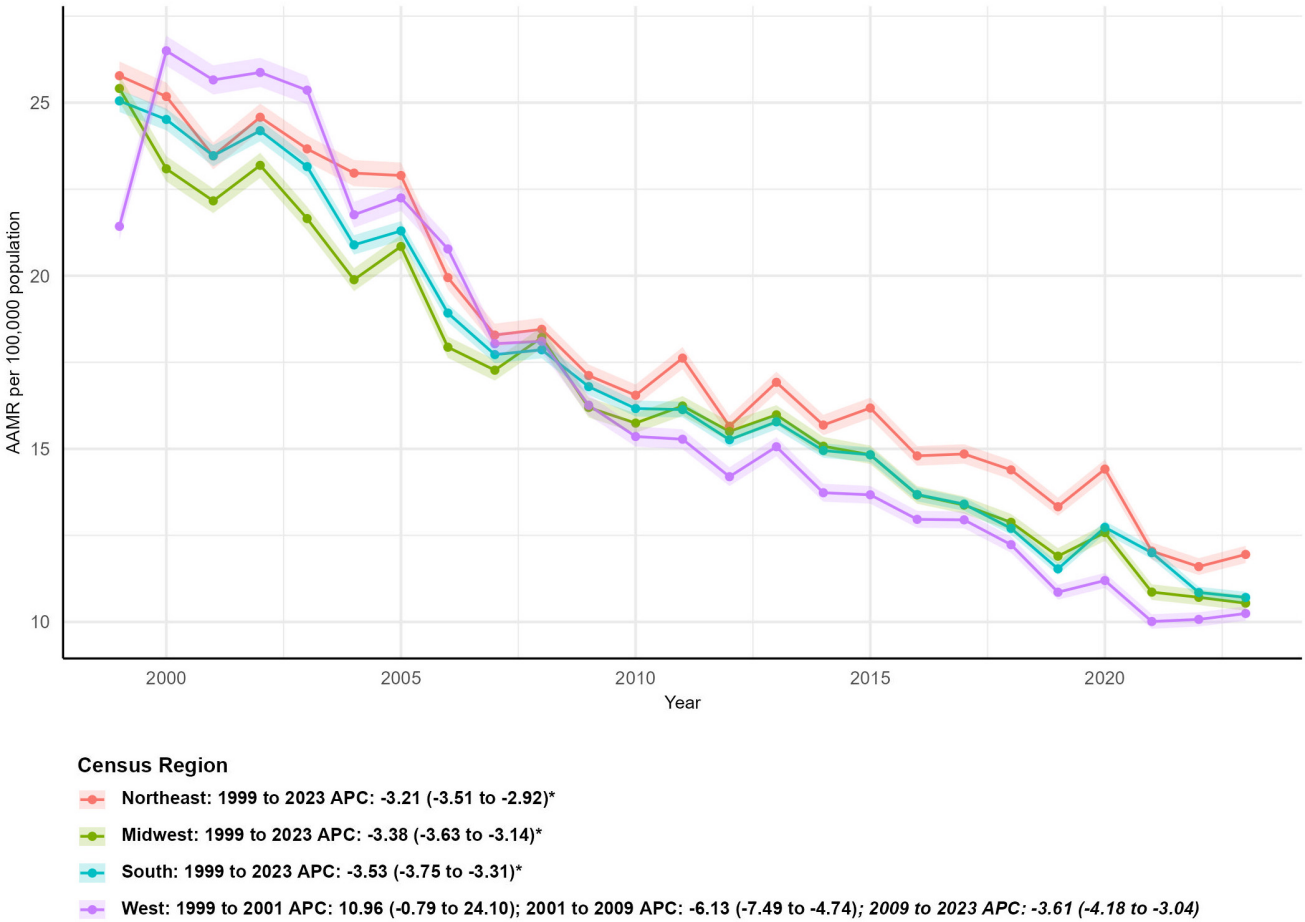
Age-adjusted mortality rates (AAMR) for asthma and pneumonia by U.S. Census Region (Northeast, Midwest, South, West) from 1999 to 2023, including region-specific average annual percent changes (AAPCs).

All four U.S. Census Regions (Northeast, Midwest, South, West) exhibited significant declines in asthma- and pneumonia-related mortality, though the magnitude of reduction varied by region (Table 1; Figure 3). The Midwest region had the largest percentage decrease in death counts (−45.51%), falling from 16,791 deaths in 1999 to 9,150 in 2023, accompanied by an AAPC of −3.38% (95% CI: −3.63 to −3.14, *P* < 0.05). The Northeast followed with a 38.50% reduction in deaths (from 14,985 to 9,216) and an AAPC of −3.21% (95% CI: −3.51 to −2.92, *P* < 0.05). The South region, which had the highest number of deaths in both 1999 (23,560) and 2023 (16,863), showed a 28.43% decrease in deaths and the steepest AAPC (−3.53%; 95% CI: −3.75 to −3.31, *P* < 0.05) among all regions. The West region had the smallest percentage reduction in deaths (−15.63%, from 11,384 in 1999 to 9,605 in 2023) and exhibited the most pronounced phase-specific trend: an initial increase in mortality from 1999 to 2001 (APC: 10.96%; 95% CI: −0.79 to 24.10, *P* > 0.05), followed by a sharp decline (APC: −6.13%; 95% CI: −7.49 to −4.74, *P* < 0.05) between 2001 and 2009, and a subsequent steady decrease (APC: −3.10%; 95% CI: −4.18 to −3.04, *P* < 0.05) from 2009 to 2023. In 2023, the West had the lowest AAMR (10.24 per 100,000; 95% CI: 10.04–10.45) across all four regions, with an AAPC of −3.33% (95% CI: −4.32 to −2.33, *P* < 0.05; Figure 3).

### 5.4 Mortality trends by race/ethnicity

**Figure 4:**
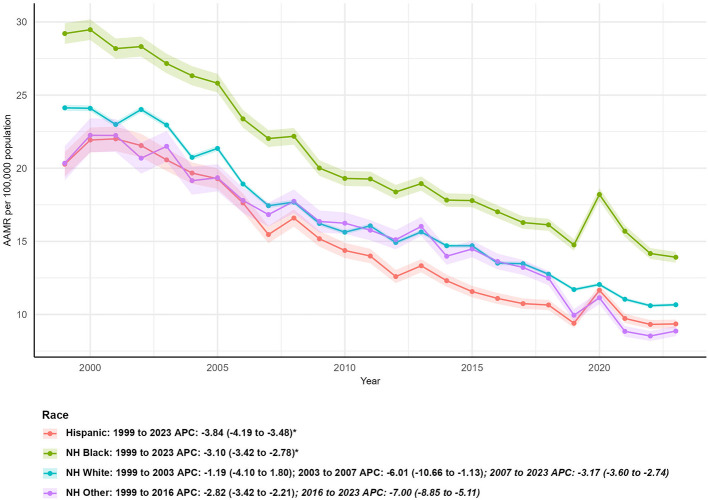
Racial/ethnic disparities in asthma and pneumonia-related age-adjusted mortality rates (AAMR) in the U.S. (1999–2023), with average annual percent changes (AAPCs) for Hispanic, Non-Hispanic (NH) Black, NH White, and NH Other populations.

Substantial disparities in mortality trends were observed across racial and ethnic groups, with both increases and decreases in death counts and consistent declines in AAMR (Table 1; Figure 4).

Hispanic population: Despite a 60.45% increase in death counts (from 2,521 in 1999 to 4,045 in 2023), the AAMR decreased from 20.28 per 100,000 (95% CI: 19.43–21.12) to 9.35 per 100,000 (95% CI: 9.06–9.65), with the steepest AAPC (−3.84%; 95% CI: −4.19 to −3.48, *P* < 0.05) among all racial/ethnic groups.

Non-Hispanic (NH) Black population: NH Black individuals had the highest AAMR in 1999 (29.21 per 100,000; 95% CI: 28.50–29.92) and 2023 (13.91 per 100,000; 95% CI: 13.55–14.28). Deaths decreased by 15.74% (from 6,863 to 5,783), and the AAPC was −3.10% (95% CI: −3.42 to −2.78, *P* < 0.05).

NH White population: This group accounted for the largest share of deaths in both 1999 (55,768) and 2023 (32,248), with a 42.17% reduction in deaths. The AAMR declined from 24.13 per 100,000 (95% CI: 23.93–24.33) to 10.67 per 100,000 (95% CI: 10.55–10.79), and the AAPC was −3.33% (95% CI: −4.24 to −2.40, *P* < 0.05).

NH Other population: This group had the largest percentage increase in death counts (99.23%, from 1,298 to 2,586) but also a significant decline in AAMR (from 20.34 per 100,000; 95% CI: 19.18–21.51 to 8.87 per 100,000; 95% CI: 8.52–9.21) and an AAPC of −4.06% (95% CI: −4.72 to −3.39, *P* < 0.05l; Figure 4).

### 5.5 Mortality trends by urbanization level

**Figure 5:**
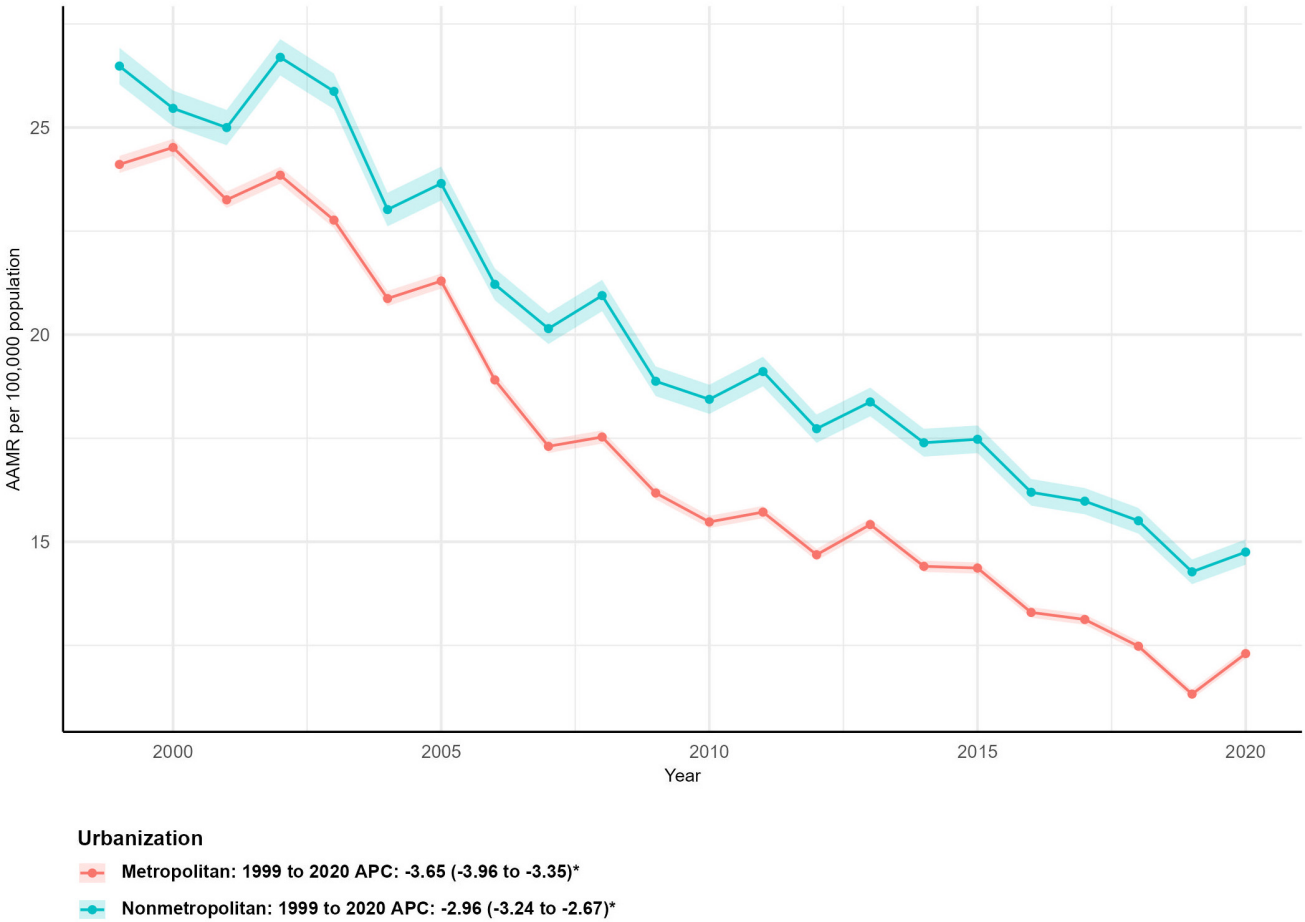
Urban-rural differences in age-adjusted mortality rates (AAMR) for asthma and pneumonia in the U.S. (1999–2020), comparing metropolitan and nonmetropolitan areas with corresponding average percent changes (APCs).

Mortality rates were consistently higher in nonmetropolitan (rural) areas compared to metropolitan (urban) areas throughout the study period (Table 1; Figure 5). In 1999, the AAMR in nonmetropolitan areas was 26.48 per 100,000 (95% CI: 26.04–26.93), vs. 24.11 per 100,000 (95% CI: 23.90–24.32) in metropolitan areas. By 2020 (the latest year with available urbanization-specific AAMR data), the AAMR in nonmetropolitan areas remained higher (14.75 per 100,000; 95% CI: 14.45–15.06) than in metropolitan areas (12.30 per 100,000; 95% CI: 12.18–12.42).

Both urbanization levels showed significant declines in mortality, but metropolitan areas had a steeper reduction: deaths in metropolitan areas decreased by 33.40% (from 52,873 in 1999 to 35,215 in 2023) with an AAPC of−3.65% (95% CI: −3.96 to −3.35, *P* < 0.05). In contrast, nonmetropolitan areas had a 30.53% reduction in deaths (from 13,847 to 9,620) and a lower AAPC of −2.96% (95% CI: −3.24 to −2.67, ^*^P^*^ < 0.05; Figure 5).

### 5.6 Mortality trends by age group

**Figure 6:**
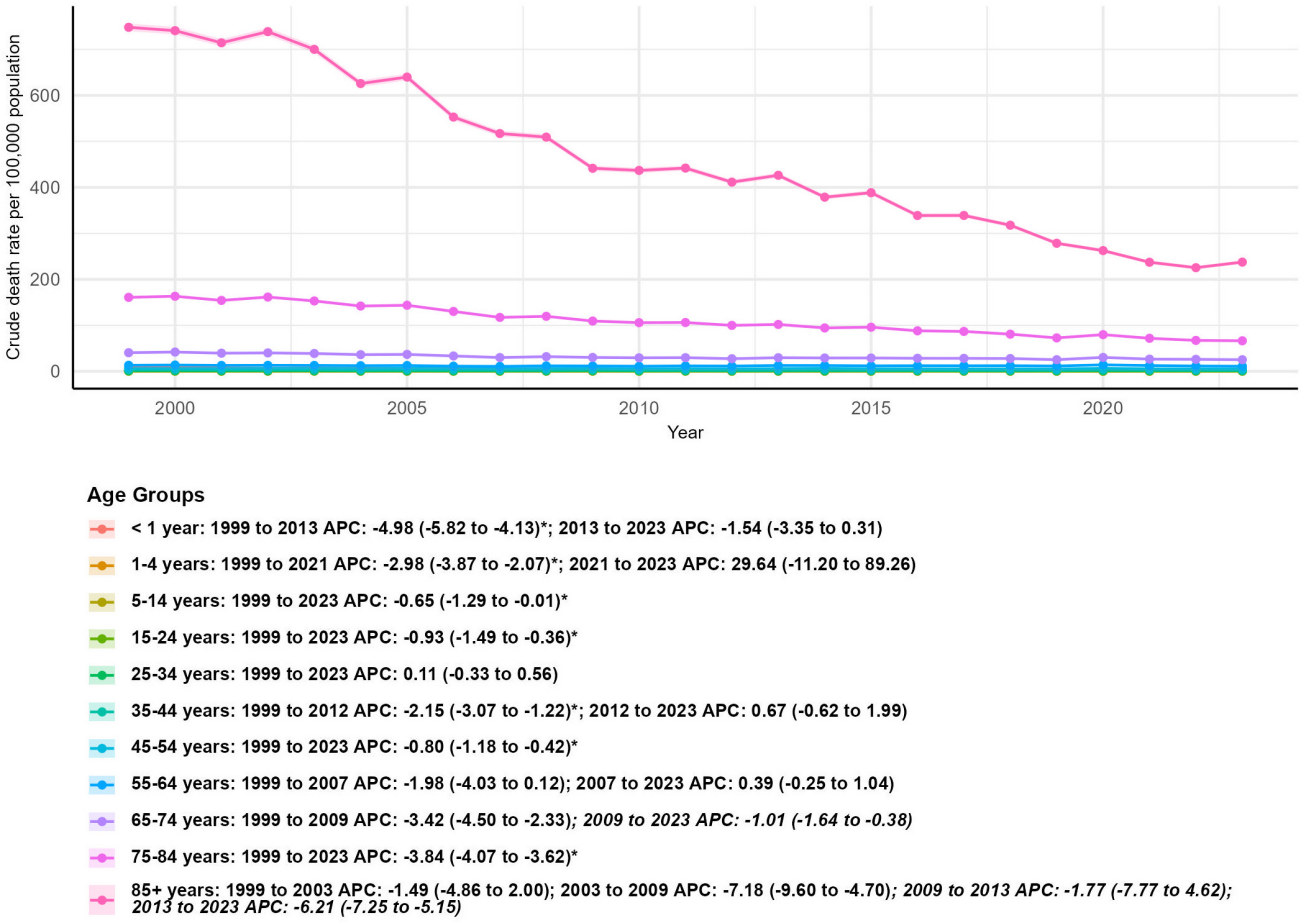
Age-stratified crude mortality rates for asthma and pneumonia in the U.S. (1999–2023), with average annual percent changes (AAPCs) for each age group (< 1 year to = 85 years).

Age was the most key factor influencing asthma-and pneumonia-related mortality, with extreme disparities between younger and older age groups (Table 1; Figure 6).

In 1999, the 85+ years age group had the highest crude mortality rate (747.90 per 100,000 population), while the 5–14 years group had the lowest (0.51 per 100,000). By 2023, the 85+ group still had the highest rate (237.48 per 100,000), though it experienced the largest percentage reduction (−52.65%) in deaths (from 31,068 to 14,712). The 85+ group also showed a dramatic AAPC of −4.95% (95% CI: −6.20 to −3.69, *P* < 0.05), (Figure 6). The < 1 year age group had the second-largest percentage reduction in deaths (−57.69%, from 312 to 132) and an AAPC of −3.56% (95% CI: −4.40 to −2.71, *P* < 0.05). However, infant mortality trends slowed after 2013, with a non-significant APC of −1.54% (95% CI: −3.35 to 0.31) compared to a steeper decline (APC: −4.98%; 95% CI: −5.82 to −4.13, *P* < 0.05) from 1999 to 2013 (Figure 6).

In contrast to older age groups, several younger and middle-age groups showed minimal declines or even increases in mortality: 1–4 years: Deaths decreased by only 12.41% (from 145 to 127), with a non-significant AAPC of −0.60% (95% CI: −3.61 to 2.49).

25–34 years: This was the only age group with an increase in deaths (+9.63%, from 592 to 649) and a non-significant AAPC of 0.11% (95% CI: −0.33 to 0.56), indicating stable mortality over the study period.

55–64 years: Deaths increased by 48.14% (from 3,137 to 4,647), and the AAPC was non-significant (−0.40%; 95% CI: −1.17 to 0.37), reflecting a plateau in mortality after 2007 (Figure 6).

5–14 and 15–24 years: Both groups had small but significant declines in AAMR, with AAPCs of −0.65% (95% CI: −1.29 to −0.01, *P* < 0.05) and −0.93% (95% CI: −1.49 to −0.36, ^*^P^*^ < 0.05), respectively. 35–44 and 45–54 years: These middle-age groups showed moderate declines, with AAPCs of −0.86% (95% CI: −1.59 to −0.13, *P* < 0.05) and −0.80% (95% CI: −1.18 to −0.42, *P* < 0.05), respectively (Figure 6).

## 6 Discussion

This study analyzed 24 years of asthma and pneumonia-related mortality data from the CDC WONDER database, revealing a 32.80% reduction in total deaths and a steady annual decline in AAMR across the United States. This downward trend aligns with two decades of U.S. public health advancements, including widespread inhaled corticosteroid use for asthma, pneumococcal/influenza vaccination campaigns, and improved acute care access for severe pneumonia (24, 25). Critically, this decline was consistent across most demographic and geographic subgroups, indicating that national interventions have delivered population-wide benefits (26). However, subgroup-specific differences in the magnitude of reduction highlight unmet needs. However, the magnitude of mortality reduction varied substantially among subgroups—such as by age, race, and region—highlighting persistent disparities that warrant targeted attention. These differences reflect underlying inequities in healthcare access, socioeconomic status, and environmental exposures, which continue to shape respiratory disease outcomes in the U.S. All associations between mortality trends and potential contributing factors discussed below are speculative. As this is a retrospective observational study, our interpretations are hypothesis-generating and do not imply definitive causal relationships.

Males consistently exhibited higher AAMRs than females (1999: 29.24 vs. 21.93 per 100,000; 2023: 12.68 vs. 9.38 per 100,000) and experienced a smaller percentage reduction in deaths (20.38 vs. 42.20% for females) over the study period. This pattern is consistent with prior research linking male sex to increased respiratory disease mortality, which may stem from biological and behavioral factors: males often have reduced lung function compared to females (27), higher rates of smoking and occupational exposure to respiratory irritants (28), and lower likelihood of seeking timely medical care for asthma or pneumonia symptoms (29). The slightly lower AAPC for males further suggests that existing interventions have not fully addressed male-specific risk factors. For example, smoking cessation programs and occupational safety regulations may need to be tailored to male-dominated industries, while public health campaigns could emphasize the importance of early care-seeking for respiratory symptoms among men.

All four U.S. Census Regions showed significant reductions in mortality, but the Midwest and Northeast outperformed the South and West. The Midwest's greater mortality reduction might be related to factors such as robust public health systems in states like Minnesota and Wisconsin—where pneumococcal vaccination rates among older adults are high and primary care networks for chronic asthma management are strong (30)—but our study cannot definitively establish this causal link. In contrast, the West experienced a smaller decline with phase-specific trends that merit closer scrutiny, including an initial non-significant increase in mortality during the early period followed by sharp declines. The West's early-period increase could potentially be associated with regional events such as wildfires—known to release particulate matter and ozone that exacerbate asthma and pneumonia (31)—though this remains a speculative association given the lack of direct wildfire exposure data in our dataset. The subsequent decline likely reflects improved wildfire smoke mitigation strategies and expanded coverage of relevant vaccines, such as pneumococcal and influenza vaccines (32). Notably, the West's 2023 AAMR was the lowest among all regions, indicating that targeted interventions can reverse regional disparities. However, the persistently small overall reduction in deaths suggests ongoing challenges. The South, despite having the highest number of deaths in both 1999 and 2023, showed a moderate decline. This may be driven by the region's high burden of poverty and chronic conditions, both of which increase susceptibility to severe asthma and pneumonia (33). Addressing these social determinants of health will be critical to accelerating reductions in mortality in the South.

Racial/ethnic subgroups exhibited striking differences in mortality trends. While all groups saw AAMR declines, NH Black individuals maintained the highest AAMRs in 1999 and 2023—a disparity rooted in systemic racism, including historical redlining and implicit bias in clinical care (34, 35). The 15.74% reduction in NH Black deaths, though significant, was smaller than that of NH White individuals, highlighting the need for anti-racist public health policies. In contrast, Hispanic and NH Other populations showed large increases in death counts but steep AAMR declines. The rising death counts likely reflect the rapid growth of these populations in the U.S. (36), while the AAMR declines suggest that existing interventions are improving outcomes. For the NH Other group (which includes Asian, Native Hawaiian, and Pacific Islander populations), the steep AAMR decline may also reflect cultural factors (37), though small sample sizes in this group warrant caution in interpretation. NH White individuals, who accounted for the majority of deaths, showed a substantial 42.17% reduction in deaths—consistent with their higher rates of healthcare access and vaccine uptake (38). However, the group's AAPC was lower than that of Hispanic and NH Other populations, suggesting that interventions for NH White individuals may be reaching a plateau.

Non-metropolitan (rural) areas consistently had higher AAMRs than metropolitan (urban) areas—with 2020 AAMRs of 14.75 vs. 12.30 per 100,000—and a smaller percentage reduction in deaths. This gap reflects rural America's unique challenges: shortages of primary care providers and specialists, longer travel times to hospitals, and higher rates of smoking and poverty (39, 40). The lower AAPC for rural areas indicates that urban-rural disparities are widening—a trend exacerbated by the COVID-19 pandemic, which strained rural healthcare systems (41). To address this, policymakers could invest in telehealth for asthma and pneumonia management and expand rural hospital funding to maintain acute care services. Community health workers, who bridge gaps between rural residents and healthcare systems, could also play a key role in increasing vaccine uptake and chronic disease management (42).

Age was the strongest predictor of mortality, with the 85+ years group showing the highest crude mortality rates in 1999 and 2023. Despite this, the 85+ group had the largest percentage reduction in deaths and the steepest AAPC—a testament to improvements in geriatric care, such as better management of comorbidities and expanded pneumococcal/influenza vaccination among nursing home residents (43, 44). In contrast, younger and middle-age groups showed minimal progress or worsening trends: The 25–34 years group was the only one with an increase in deaths, with a non-significant AAPC. This may reflect rising rates of risk factors like vaping (which exacerbates asthma) and mental health conditions (e.g., depression, which reduces adherence to asthma medications) (45, 46). The 55–64 years group had a 48.14% increase in deaths and a non-significant AAPC, likely driven by the growing prevalence of obesity and diabetes in this cohort—both of which increase pneumonia severity (47). The 1–4 years group had a small death reduction and non-significant AAPC, highlighting unmet needs in pediatric asthma care (48). These trends suggest that public health efforts have disproportionately focused on older adults—while younger and middle-age groups require more targeted interventions, such as youth vaping prevention programs and chronic disease management for middle-aged adults.

## 7 Strengths and limitations

This research utilizes a dataset that is nationally representative and covers a period of 24 years, providing an in-depth examination of trends related to mortality caused by asthma and pneumonia. One of the principal advantages of the study is its stratification of data based on demographic and geographic parameters, such as race, age, gender, and distinctions between urban and rural environments. This stratified methodology improves the significance and precision of the results by considering risk factors specific to populations and regional differences, consequently reducing the influence of discrepancies in patient demographics and access to healthcare services.

Our study has several limitations. First, a major limitation of this study is the lack of ACS-level data on SES, insurance coverage, HPSA status, and MUA/P designations—factors known to influence healthcare access and respiratory disease outcomes. It has the potential for ecological fallacy. Second, 2023 AAMR data for urban/rural areas was replaced with 2020 data, which may not reflect recent trends. Third, unmeasured variables may confound observed disparities. While age-adjustment mitigated some confounding, residual bias from unmeasured factors cannot be fully excluded. Besides, de-identified public use data excludes individual-level clinical details (e.g., asthma control status, pneumonia etiologic agents), limiting analysis of disease-specific risk factors. Additionally, AAMRs were not available for age stratification, which may introduce bias due to differences in population age structure across subgroups.

## 8 Conclusion

From 1999 to 2023, the U.S. achieved significant progress in reducing asthma- and pneumonia-related mortality, driven by advancements in vaccination, chronic disease management, and public health policy. However, persistent disparities across sex, race, geography, and age underscore the need for equitable, targeted interventions—particularly those addressing social determinants of health.

To address these disparities, specific policy and intervention examples include: (1) Healthcare access: Expand telehealth services for rural and underserved populations to improve asthma monitoring and pneumonia follow-up care; increase funding for community health centers in low-income areas to reduce care delays. (2) Vaccination equity: Implement targeted outreach programs in rural and minority communities to improve pneumococcal and influenza vaccine uptake, which remains 10%−15% lower in these groups compared to the general population. (3) Behavioral and environmental interventions: Launch youth vaping prevention campaigns in schools to address rising asthma exacerbation risks in 25–34-year-olds; enforce stricter air quality standards in areas with high wildfire or industrial pollution to reduce respiratory irritant exposure. (4) Anti-racist policies: Train healthcare providers on implicit bias to reduce disparities in asthma/pneumonia treatment for Non-Hispanic Black individuals; address historical redlining's legacy by investing in healthcare infrastructure in segregated neighborhoods.

Future research should: (1) Link mortality data to clinical records to analyze disease-specific factors (e.g., asthma control status, antibiotic resistance in pneumonia); (2) Evaluate the long-term impact of COVID-19 on asthma/pneumonia mortality, particularly in rural areas; (3) Explore racial/ethnic disparities in treatment adherence and healthcare utilization to inform culturally competent interventions. By centering equity and addressing social determinants of health, policymakers and public health practitioners can accelerate progress toward eliminating respiratory disease mortality disparities in the U.S.

## Data Availability

The datasets presented in this study can be found in online repositories. The names of the repository/repositories and accession number(s) can be found below: the datasets generated and/or analysed during the current study are available in the CDC wonder database. The datasets generated and/or analysed during the current study are available on CDC wonder database [https://wonder.cdc.gov/mcd-icd10.html].

## References

[1] GBD 2016 Lower Respiratory Infections Collaborators. Estimates of the global, regional, and national morbidity, mortality, and aetiologies of lower respiratory infections in 195 countries, 1990-2016: a systematic analysis for the global burden of disease study 2016. Lancet Infect Dis. (2018) 18:1191–210. doi: 10.1016/S1473-3099(18)30310-4 30243584 PMC6202443

[2] Global Initiative for Asthma (GINA). GINA 2024 Global Strategy for Asthma Management and Prevention. (2024). Available online at: https://ginasthma.org/gina-2024-global-strategy-for-asthma-management-and-prevention/ (Accessed October 10, 2025).

[3] Mandell LA Wunderink RG Anzueto A Bartlett JG Campbell GD Dean NC . Infectious Diseases Society of America/American Thoracic Society consensus guidelines on the management of community-acquired pneumonia in adults. Clin Infect Dis. (2007) 44 Suppl 2:S27–72. doi: 10.1086/511159 17278083 PMC7107997

[4] GBD 2019 Chronic Respiratory Diseases Collaborators. Global burden of chronic respiratory diseases and risk factors, 1990-2019: an update from the global burden of disease study 2019. EClinicalMedicine. (2023) 59:101936. doi: 10.1016/j.eclinm.2023.101936 37229504 PMC7614570

[5] Terraneo S Polverino E Cilloniz C Amaro R Vennera M del C Gabarrus A . Severity and outcomes of community acquired pneumonia in asthmatic patients. Respir Med. (2014) 108:1713–22. doi: 10.1016/j.rmed.2014.09.001 25245791

[6] Djeddi S Fernandez-Salinas D Huang GX Aguiar VRC Mohanty C Kendziorski C . Rhinovirus infection of airway epithelial cells uncovers the non-ciliated subset as a likely driver of genetic risk to childhood-onset asthma. Cell Genom. (2024) 4:100636. doi: 10.1016/j.xgen.2024.100636 39197446 PMC11480861

[7] Kravchenko J Akushevich I Abernethy AP Holman S Ross WG Jr Lyerly HK. Long-term dynamics of death rates of emphysema, asthma, and pneumonia and improving air quality. Int J Chron Obstruct Pulmon Dis. (2014) 9:613–27. doi: 10.2147/COPD.S59995 25018627 PMC4075234

[8] Centers for Disease Control and Prevention (CDC). National Asthma Statistics. (2023). Available online at: https://www.cdc.gov/asthma/statistics/index.htm. (Accessed October 10, 2025).

[9] Akinbami LJ Moorman JE Liu X. Asthma prevalence, health care use, and mortality: United States, 2005-2009. Natl Health Stat Report. (2011) 35:1–14. doi: 10.1542/peds.110.2.315 21355352

[10] Centers for Disease Control and Prevention (CDC). Pneumonia Surveillance Report. (2023). Available online at: https://www.cdc.gov/pneumococcal/surveillance/index.htm (Accessed October 10, 2025).

[11] Eshwara VK Mukhopadhyay C Rello J. Community-acquired bacterial pneumonia in adults: an update. Indian J Med Res. (2020) 151:287–302. doi: 10.4103/ijmr.IJMR_1678_19 32461392 PMC7371062

[12] Natarajan A Shetty A Delanerolle G Zeng Y Zhang Y Raymont V . A systematic review and meta-analysis of long COVID symptoms. Syst Rev. (2023) 12:88. doi: 10.1186/s13643-023-02250-0 37245047 PMC10220332

[13] Safiabadi Tali SH LeBlanc JJ Sadiq Z Oyewunmi OD Camargo C Nikpour B . Tools and techniques for severe acute respiratory syndrome coronavirus 2 (SARS-CoV-2)/COVID-19 detection. Clin Microbiol Rev. (2021) 34:e00228-20. doi: 10.1128/CMR.00228-20 33980687 PMC8142517

[14] Nowalk MP Wateska AR Lin CJ Schaffner W Harrison LH Zimmerman RK . Racial disparities in adult pneumococcal vaccination indications and pneumococcal hospitalizations in the US. J Natl Med Assoc. (2019) 111:540–5. doi: 10.1016/j.jnma.2019.04.011 31171344 PMC6888932

[15] Garcia-Garcia ML Calvo Rey C Del Rosal Rabes T. Pediatric asthma and viral infection. Asma y virus en el niño Arch Bronconeumol. (2016) 52:269–73. doi: 10.1016/j.arbres.2015.11.008 26766408 PMC7131251

[16] Huang K Yang T Xu J Yang L Zhao J Zhang X . Prevalence, risk factors, and management of asthma in China: a national cross-sectional study. Lancet. (2019) 394:407–18. doi: 10.1016/S0140-6736(19)31147-X 31230828

[17] Raja AR Ghori FF Zaide DB Zubairi ABS. Demographic and regional trends in asthma mortality in the United States, 1999-2020. Expert Rev Respir Med. (2025) 19:399–405. doi: 10.1080/17476348.2025.2474140 40022292

[18] Pennington E Yaqoob ZJ Al-Kindi SG Zein J. Trends in asthma mortality in the United States: 1999 to 2015. Am J Respir Crit Care Med. (2019) 199:1575–7. doi: 10.1164/rccm.201810-1844LE 30917289 PMC6835077

[19] Kilpatrick K Ambrose CS Lindsley AW Oppenheimer J. At-home asthma mortality unchanged despite declining mortality in other settings: US death certificate data (2000-2019). Ann Allergy Asthma Immunol. (2024) 132:216–22. doi: 10.1016/j.anai.2023.10.009 37848103

[20] Kodadhala V Obi J Wessly P Mehari A Gillum RF. Asthma-related mortality in the United States, 1999 to 2015: a multiple causes of death analysis. Ann Allergy Asthma Immunol. (2018) 120:614–9. doi: 10.1016/j.anai.2018.03.005 29548908

[21] Kearney GD Wooten W Mohan A Christopher Carter J Jones K Blount T . Asthma deaths in North Carolina: 1999-2016. J Asthma. (2020) 57:478–86. doi: 10.1080/02770903.2019.1579830 30810458

[22] Ingram DD Franco SJ. 2013 NCHS urban-rural classification scheme for counties. Vital Health Stat 2. (2014) 165:1–73. 24776070

[23] Anderson RN Rosenberg HM. Age standardization of death rates: implementation of the year 2000 standard. Natl Vital Stat Rep. (1998) 47:1–20. 9796247

[24] Expert Expert Panel Working Group of the National Heart Lung and Blood Institute (NHLBI) Administered and Coordinated National Asthma Education and Prevention Program Coordinating Committee (NAEPPCC) Cloutier MM Baptist AP Blake KV . 2020 Focused updates to the asthma management guidelines: a report from the national asthma education and prevention program coordinating committee expert panel working group. J Allergy Clin Immunol. (2020) 146:1217–70. doi: 10.1016/j.jaci.2020.10.003 33280709 PMC7924476

[25] Wyplosz B Fernandes J Sultan A Roche N Roubille F Loubet P . Pneumococcal and influenza vaccination coverage among at-risk adults: a 5-year French national observational study. Vaccine. (2022) 40:4911–21. doi: 10.1016/j.vaccine.2022.06.071 35811205

[26] Pope CA 3rd Ezzati M Dockery DW. Fine-particulate air pollution and life expectancy in the United States. N Engl J Med. (2009) 360:376–86. doi: 10.1056/NEJMsa0805646 19164188 PMC3382057

[27] Heraganahally SS Howarth T Sorger L Ben Saad H. Sex differences in pulmonary function parameters among Indigenous Australians with and without chronic airway disease. PLoS ONE. (2022) 17:e0263744. doi: 10.1371/journal.pone.0263744 35134094 PMC8824342

[28] Xu ZY Blot WJ Xiao HP Wu A Feng YP Stone BJ . Smoking, air pollution, and the high rates of lung cancer in Shenyang, China. J Natl Cancer Inst. (1989) 81:1800–6. doi: 10.1093/jnci/81.23.1800 2555531

[29] Silveyra P Fuentes N Rodriguez Bauza DE. Sex and gender differences in lung disease. Adv Exp Med Biol. (2021) 1304:227–58. doi: 10.1007/978-3-030-68748-9_14 34019273 PMC8221458

[30] Lu PJ Nuorti JP. Uptake of pneumococcal polysaccharide vaccination among working-age adults with underlying medical conditions, United States, 2009. Am J Epidemiol. (2012) 175:827–37. doi: 10.1093/aje/kwr376 22403807

[31] Heaney A Stowell JD Liu JC Basu R Marlier M Kinney P. Impacts of fine particulate matter from wildfire smoke on respiratory and cardiovascular health in California. Geohealth. (2022) 6:e2021GH000578. doi: 10.1029/2021GH000578 35795228 PMC9166629

[32] Wilgus ML Merchant M. Clearing the air: understanding the impact of wildfire smoke on asthma and COPD. Healthcare. (2024) 12:307. doi: 10.3390/healthcare12030307 38338192 PMC10855577

[33] Torres A Blasi F Dartois N Akova M. Which individuals are at increased risk of pneumococcal disease and why? Impact of COPD, asthma, smoking, diabetes, and/or chronic heart disease on community-acquired pneumonia and invasive pneumococcal disease. Thorax. (2015) 70:984–9. doi: 10.1136/thoraxjnl-2015-206780 26219979 PMC4602259

[34] Geronimus AT Hicken M Keene D Bound J. “Weathering” and age patterns of allostatic load scores among blacks and whites in the United States. Am J Public Health. (2006) 96:826–33. doi: 10.2105/AJPH.2004.060749 16380565 PMC1470581

[35] Institute Institute of Medicine (US) Committee on Understanding Eliminating Racial Ethnic Disparities in Health Care Smedley BD Stith AY Nelson AR . Unequal Treatment: Confronting Racial and Ethnic Disparities in Health Care. Washington (DC): National Academies Press (US) (2003). 25032386

[36] Baral N Jabbar ABA Noor A Mirza M DeVrieze B Hildenbrand A . Demographic and geographical trends in chronic lower respiratory diseases mortality in the United States, 1999 to 2020. Respir Res. (2024) 25:258. doi: 10.1186/s12931-024-02880-5 38915019 PMC11197268

[37] Ra CK Pehlivan N Kim H Sussman S Unger JB Businelle MS. Smoking prevalence among Asian Americans: associations with education, acculturation, and gender. Prev Med Rep. (2022) 30:102035. doi: 10.1016/j.pmedr.2022.102035 36531113 PMC9747624

[38] Kolobova I Nyaku MK Karakusevic A Bridge D Fotheringham I O'Brien M. Vaccine uptake and barriers to vaccination among at-risk adult populations in the US. Hum Vaccin Immunother. (2022) 18:2055422. doi: 10.1080/21645515.2022.2055422 35536017 PMC9248946

[39] Patrick DL Stein J Porta M Porter CQ Ricketts TC. Poverty, health services, and health status in rural America. Milbank Q. (1988) 66:105–36. doi: 10.2307/3349987 3262817

[40] Raju S Keet CA Paulin LM Matsui EC Peng RD Hansel NN . Rural residence and poverty are independent risk factors for chronic obstructive pulmonary disease in the United States. Am J Respir Crit Care Med. (2019) 199:961–9. doi: 10.1164/rccm.201807-1374OC 30384774 PMC6467317

[41] Ataç Ö Peterson LE Waters TM. The impact of the COVID-19 pandemic on vaccinations in United States primary care practices. PLoS ONE. (2025) 20:e0325934. doi: 10.1371/journal.pone.0325934 40493658 PMC12151362

[42] Berini CR Bonilha HS Simpson AN. Impact of community health workers on access to care for rural populations in the United States: a systematic review. J Community Health. (2022) 47:539–53. doi: 10.1007/s10900-021-01052-6 34817755

[43] Hoben M Hogan DB Poss JW Gruneir A McGrail K Griffith LE . Comparing quality of care outcomes between assisted living and nursing homes before and during the COVID-19 pandemic. J Am Geriatr Soc. (2023) 71:3467–79. doi: 10.1111/jgs.18499 37428008

[44] Bardenheier B Wortley P Shefer A McCauley MM Gravenstein S. Racial inequities in receipt of influenza vaccination among nursing home residents in the United States, 2008-2009: a pattern of low overall coverage in facilities in which most residents are black. J Am Med Dir Assoc. (2012) 13:470–6. doi: 10.1016/j.jamda.2012.02.001 22420974 PMC4554484

[45] Lyzwinski LN Naslund JA Miller CJ Eisenberg MJ. Global youth vaping and respiratory health: epidemiology, interventions, and policies. NPJ Prim Care Respir Med. (2022) 32:14. doi: 10.1038/s41533-022-00277-9 35410990 PMC9001701

[46] Morales-Raveendran E Goodman E West E Cone JE Katz C Weiss J . Associations between asthma trigger reports, mental health conditions, and asthma morbidity among world trade center rescue and recovery workers. J Asthma. (2019) 56:833–40. doi: 10.1080/02770903.2018.1502300 30073876

[47] Fisher-Hoch SP Mathews CE McCormick JB. Obesity, diabetes and pneumonia: the menacing interface of non-communicable and infectious diseases. Trop Med Int Health. (2013) 18:1510–9. doi: 10.1111/tmi.12206 24237786

[48] Telzak A Fiori KP Chambers EC Haughton J Levano S Reznik M. Unmet social needs and pediatric asthma severity in an urban primary care setting. Acad Pediatr. (2023) 23:1361–7. doi: 10.1016/j.acap.2023.02.009 36858248 PMC11357840

